# Supplementary material for: Day length regulates gonadotrope proliferation and reproduction via an intra-pituitary pathway in the model vertebrate Oryzias latipes
Source: Commun Biol. 2024 Mar 30;7:388. doi: 10.1038/s42003-024-06059-y (PMC10980775; doi:10.1038/s42003-024-06059-y)
Supplement: Supplementary file 2 — Supplementary information [file 42003_2024_6059_MOESM2_ESM.pdf]

Supporting Information for
Day length regulates gonadotrope proliferation and reproduction via an intra-pituitary pathway in the model vertebrate *Oryzias latipes*

Muhammad Rahmad Royan, Kjetil Hodne, Rasoul Nourizadeh-Lillabadi, Finn-Arne Weltzien, Christiaan Henkel, Romain Fontaine*

*Corresponding author: Romain Fontaine
Email: romain.fontaine@nmbu.no

This PDF file includes:

Supplementary Figure 1 to 8

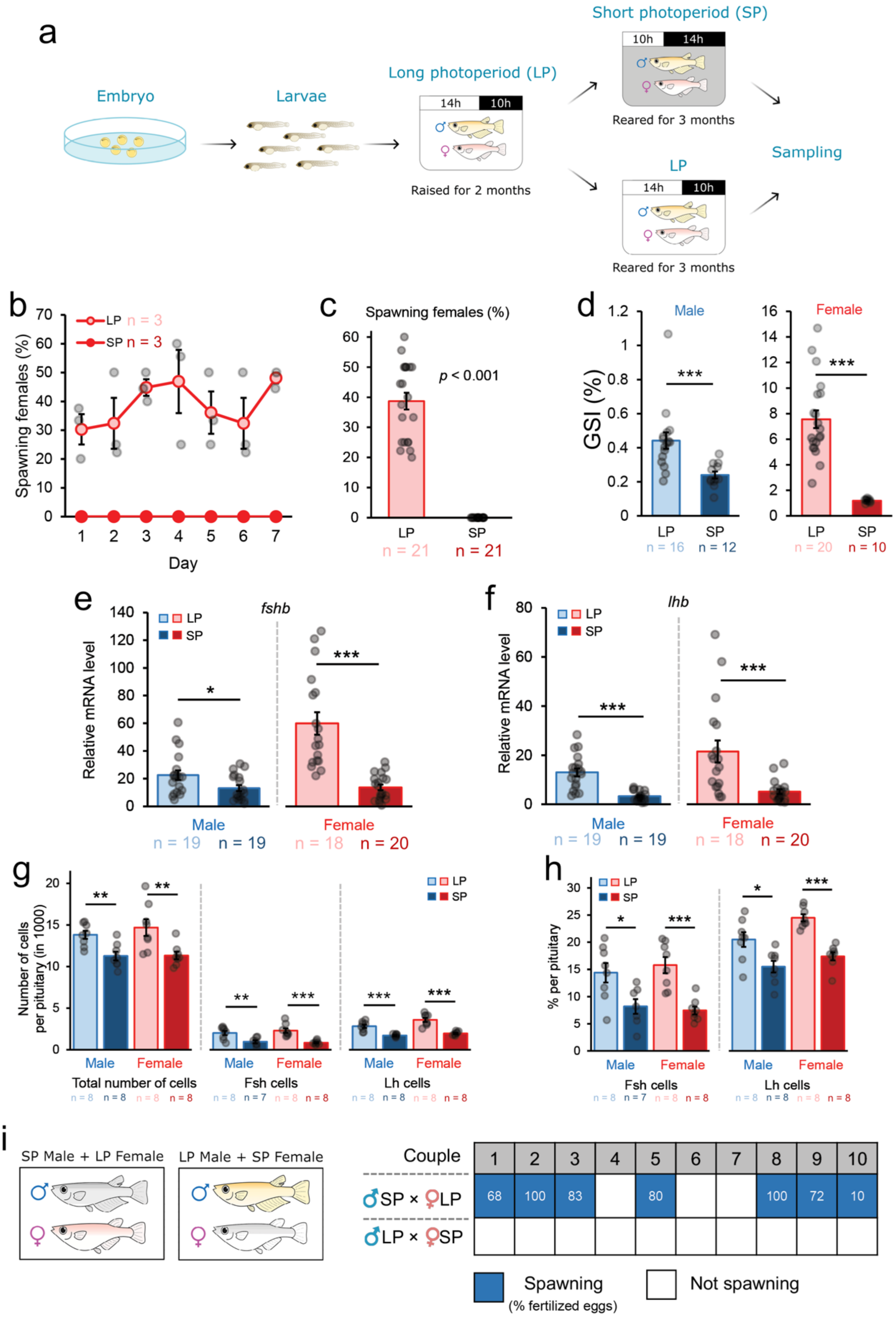
a
Embryo
Larvae
Long photoperiod (LP)
14h 10h
Raised for 2 months
Short photoperiod (SP)
10h 14h
Reared for 3 months
LP
14h 10h
Reared for 3 months
Sampling
b
LP n = 3
SP n = 3
Spawning females (%)
Day
c
Spawning females (%)
p < 0.001
LP SP
n = 21 n = 21
d
GSI (%)
Male
Female
***
LP SP
n = 16 n = 12
n = 20 n = 10
e
fshb
Relative mRNA level
LP
SP
Male
Female
n = 19 n = 19
n = 18 n = 20
f
lhb
Relative mRNA level
Male
Female
n = 19 n = 19
n = 18 n = 20
g
Number of cells per pituitary (in 1000)
Total number of cells
Fsh cells
Lh cells
h
% per pituitary
Fsh cells
Lh cells
i
SP Male + LP Female
LP Male + SP Female
Couple 1 2 3 4 5 6 7 8 9 10
♂SP × ♀LP 68 100 83 80 100 72 10
♂LP × ♀SP
Spawning (% fertilized eggs)
Not spawning

Supplementary Figure 1. Long photoperiod stimulates reproduction by increasing gonadotrope cell activity (hormone synthesis) and cell number. (a) Illustration of the experimental protocol used. Two-month-old medaka fish raised in long photoperiod (LP) were divided into two groups reared under long (LP) and short (SP) photoperiod for 3 months before sampling. (b) Graph showing the daily percentage of spawning females kept in SP and LP during 7-day observation. (c) Graph showing the average daily percentage of spawning females kept in SP and LP for 7 consecutive days. (d) Gonadosomatic index (GSI) in male and female medaka kept in SP and LP. (e-f) Graph presenting the relative mRNA levels of *fshb* and *lhb* in the pituitary of male and female medaka in SP and LP. (g-h) Graph presenting the absolute (g) and relative (h) number of gonadotrope cells per pituitary in SP and LP fish. (i) Spawning observation in SP fish coupled with LP fish to evaluate reproductive capacity of SP fish. Number in the square indicates the percentage of fertilized eggs. Individual numbers (n) are annotated throughout the figure. Statistical analyses were performed using two-sample independent t-test (h) or Mann Whitney U test (c, d, e, f, g), in which the graph was provided as mean ± SEM while the jittered dots represent each individual (* < 0.05; ** < 0.01; *** < 0.001)

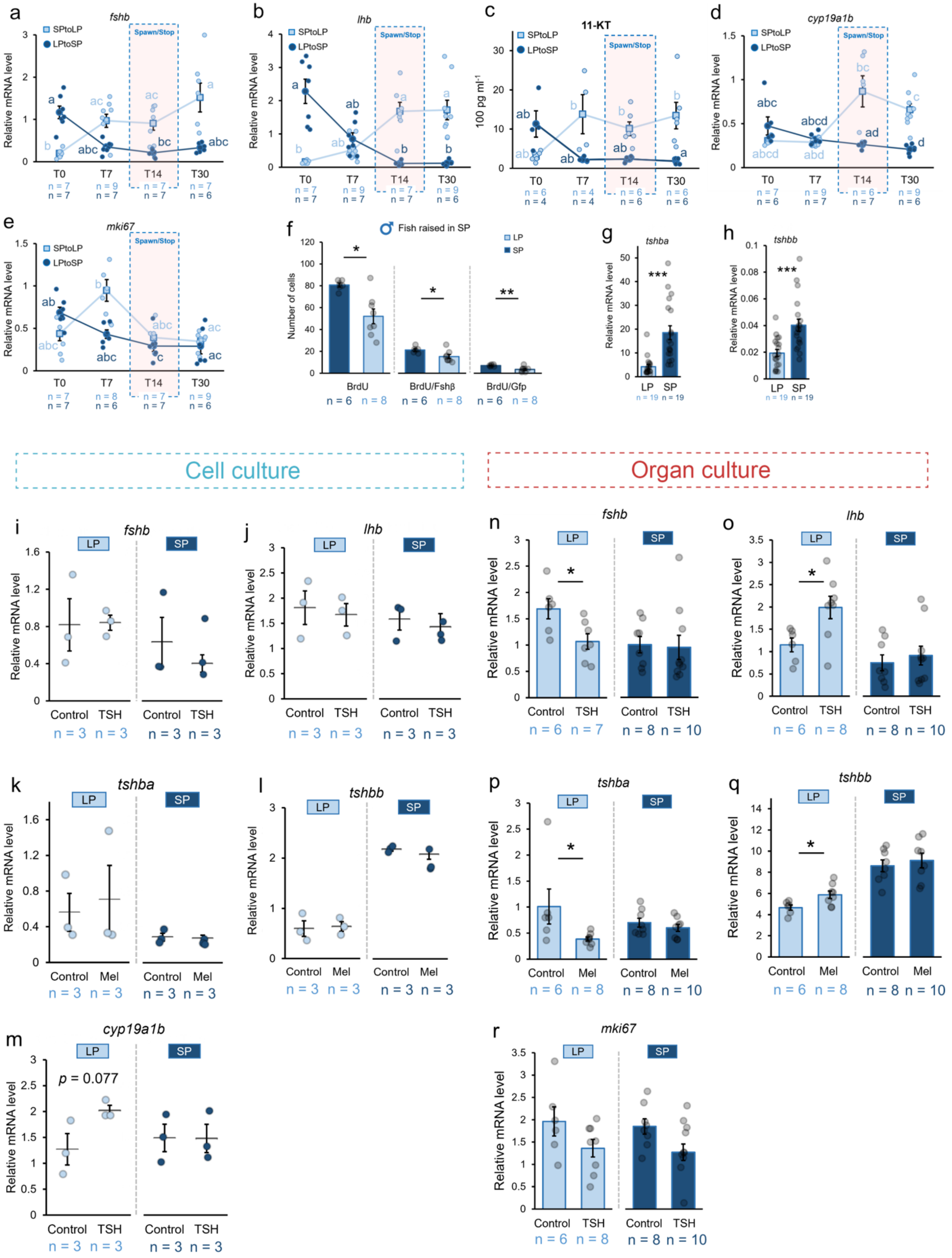
a
fshb
SPtoLP
LPtoSP
Spawn/Stop
Relative mRNA level
T0
T7
T14
T30
b
lhb
c
11-KT
100 pg ml-1
d
cyp19a1b
e
mki67
f
Fish raised in SP
LP
SP
Number of cells
BrdU
BrdU/Fshβ
BrdU/Gfp
g
tshba
h
tshbb
Cell culture
Organ culture
i
fshb
j
lhb
k
tshba
l
tshbb
m
cyp19a1b
p = 0.077
n
fshb
o
lhb
p
tshba
q
tshbb
r
mki67
Control
TSH
Mel

Supplementary Figure 2. Effect of photoperiod, Tsh and melatonin in males.

(a-e) The fluctuation of *fshb*, *lhb*, *cyp19a1b*, and *mki67* expression as well as 11-KT levels observed in males at different time points following the photoperiod change. Black asterisks display statistically differences between the SPtoLP group and the SPtoLP group for each observation day. The letters (a, b) show statistically differences between each time points and T0 which is taken as reference within one treatment group. The statistical analyses were performed using two-sample independent t-test or Mann-Whitney U test (* < 0.05; ** < 0.01; *** < 0.001). Like in females, *fshb* and *lhb* increase in SPtoLP while decrease in LPtoSP males. Significant changes are already observed for both *fshb* ($p_{SPtoLP} = 0.039$; $p_{LPtoSP} = 0.006$) and *lhb* ($p_{SPtoLP} = 0.020$; $p_{LPtoSP} < 0.001$) after 7-14 days. In line with gonadotropin mRNA levels, 11-keto testosterone (11-KT) levels also change in 7 days, increasing in SPtoLP while decreasing in LPtoSP males. Also, similar to in females, the mRNA levels for aromatase (*cyp19a1b*) show the same pattern that gonadotropins and sex steroids levels, suggesting that the same mechanisms take place in males and females.

While *mki67* levels increase in SPtoLP males, the total number of mitotic cells as well as the number of both Lh and Fsh gonadoaropes dividing (f) in SP fish exposed to LP for 5 days, are lower in males moved from SP to LP than in males kept in SP ($p_{total} = 0.024$; $p_{Fsh\ cell} = 0.024$; $p_{Lh\ cell} = 0.004$). These results suggest that opposite to in females, LP inhibits gonadotrope cell proliferation in males. Because of the number of gonadotropes and percentage of cells they represent in the pituitary is higher in LP males than in SP males, our results therefore suggest than another mechanism is playing a role in gonadotrope cell proliferation in males under LP conditions.

Tshba (g) and *tshbb* (h) mRNA levels in LP and SP condition in male medaka. As in females, *tshbb* levels are reduced in LP males compared to SP males. In contrast, the levels of *tshba* show sexual dimorphism with LP males showing lower *tshba* levels than SP males.

Effect of 0.5 µM bovine TSH pituitary extract, 10 µM melatonin, or vehicle (control) on *fshb*, *lhb*, *cyp19a1b*, *tshba*, and *tshbb* mRNA levels in dispersed pituitary cell cultures (i-m) or *ex vivo* medaka pituitary organ cultures (n-r) in male medaka from LP and SP condition (n = 3; in which each replicate represents 4 pooled pituitaries for *in vitro* studies). The statistical analyses were performed using two-sample independent t-test or Mann Whitney U test (* < 0.05; *** < 0.001). We found that similar to in females, TSH has an effect on gonadotropin mRNA levels in *ex vivo* pituitary cultures but not in dissociated pituitary cell cultures. Surprisingly, TSH stimulates *lhb* but inhibits *fshb* levels. Interestingly, the effects on gonadotropins can only be observed In LP males, and not SP males. Aromatase mRNA levels increased at nearly significance levels in LP male cell cultures following TSH stimulation. Together, these results suggest that, as in females, TSH regulate gonadotropin mNRA synthesis in males, but in an opposite manner than females for *fshb*. As in females, while no effects were observed in dissociated cell cultures, *tshba* and *tshbb* levels were regulated by melatonin in *ex vivo* pituitary organ cultures. Also similar to in females, melatonin increased *tshbb* ($p = 0.022$) and reduced *tshba* ($p = 0.020$) levels in LP males. It is surprising that, melatonin inhibits *tshba* in males, as SP males which are expected to have higher melatonin levels were found to possess higher *tshba* levels than LP males. These results thus suggest that another factor plays a more important role in the regulation of *tshba* in SP males. Nevertheless, the higher levels of *tshba* in SP males (compared to in LP males), are in line with the inhibitory effect of TSH observed on *fshb* and the lower levels of *fshb* ($p = 0.023$) observed in SP males (compared to in LP males). Finally, in contrast to in females, TSH had no effects on *mki67* levels, supporting the hypothesis that photoperiod does not regulate gonadotrope cell proliferation via Tsh in the male pituitary, and thus that another mechanism is taking place. All the graphs are represented as mean ± SEM. Individual numbers (n) are annotated throughout the figure.

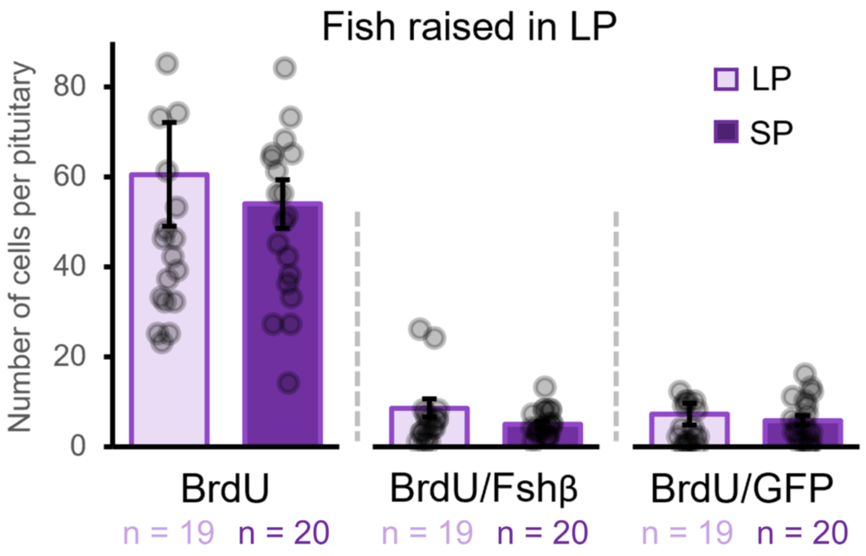

Supplementary Figure 3. Number of proliferative gonadotropes. No change in the number of mitotic cells when LP raised fish exposed to SP. Number of mitotic cells 5 days after the photoperiod regime change in mixed-sex groups. The statistical analyses were performed using two-sample independent t-test, in which the graph represents mean ± SEM while the jittered dots represent each individual. Individual numbers (n) are annotated in the figure.

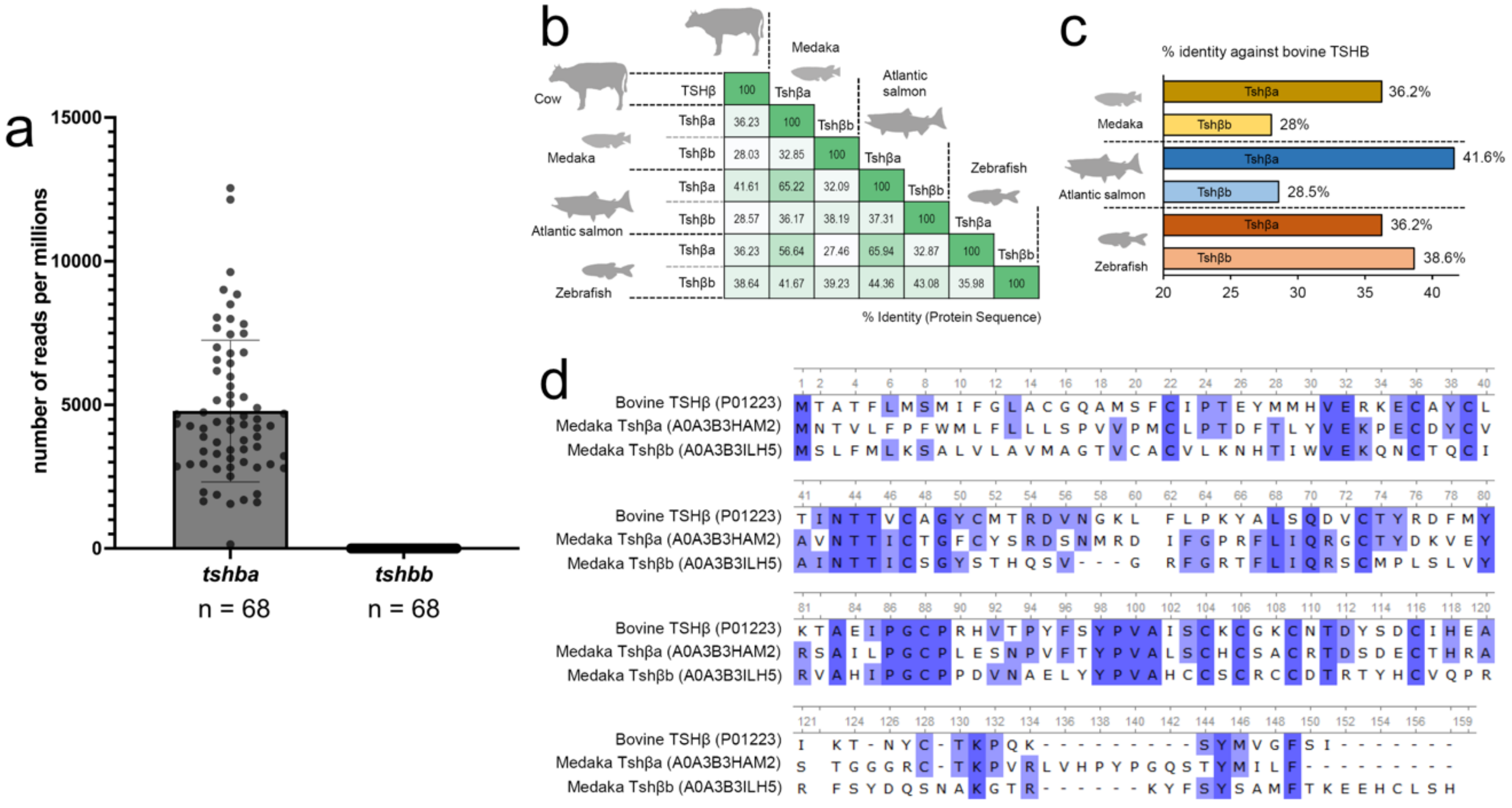

Supplementary Figure 4. Protein sequence identity of bovine TSHβ shows a more similarity to medaka Tshβa than Tshβb. (a) Number of reads of *tshba* and *tshbb* in from the medaka pituitary RNAseq dataset from both sexes combined. The graph represents mean ± SEM while the jittered dots represent each individual. Individual numbers (n) are annotated in the figure. (b) Percentage of protein sequence identity of bovine TSHβ (P01223) and other fish species, including medaka (*Oryzias latipes*; Tshβa: A0A3B3HAM2; Tshβb: A0A3B3ILH5), Atlantic salmon (*Salmo salar*; Tshβa: O73824; Tshβb: A0A1S3MIK7), and zebrafish (*Danio rerio*, Tshβa: A0A8M9PLW7; Tshβb: A0A8M6Z4G8). (c) Percentage of protein sequence identity of bovine TSHβ to medaka, Atlantic salmon, and zebrafish Tshβa and Tshβb. (d) Sequence alignment of medaka Tshβa and Tshβb to bovine TSHβ as the reference sequence showing that even if some regions seem well conserved between the mammalian TSHβ and the medaka Tshβa and Tshβb protein sequences, they still are strongly divergent. Sequences were retrieved from UniProt, while percentage of sequence identity and alignment was performed using default settings in UniProt using Clustal Omega algorithm.

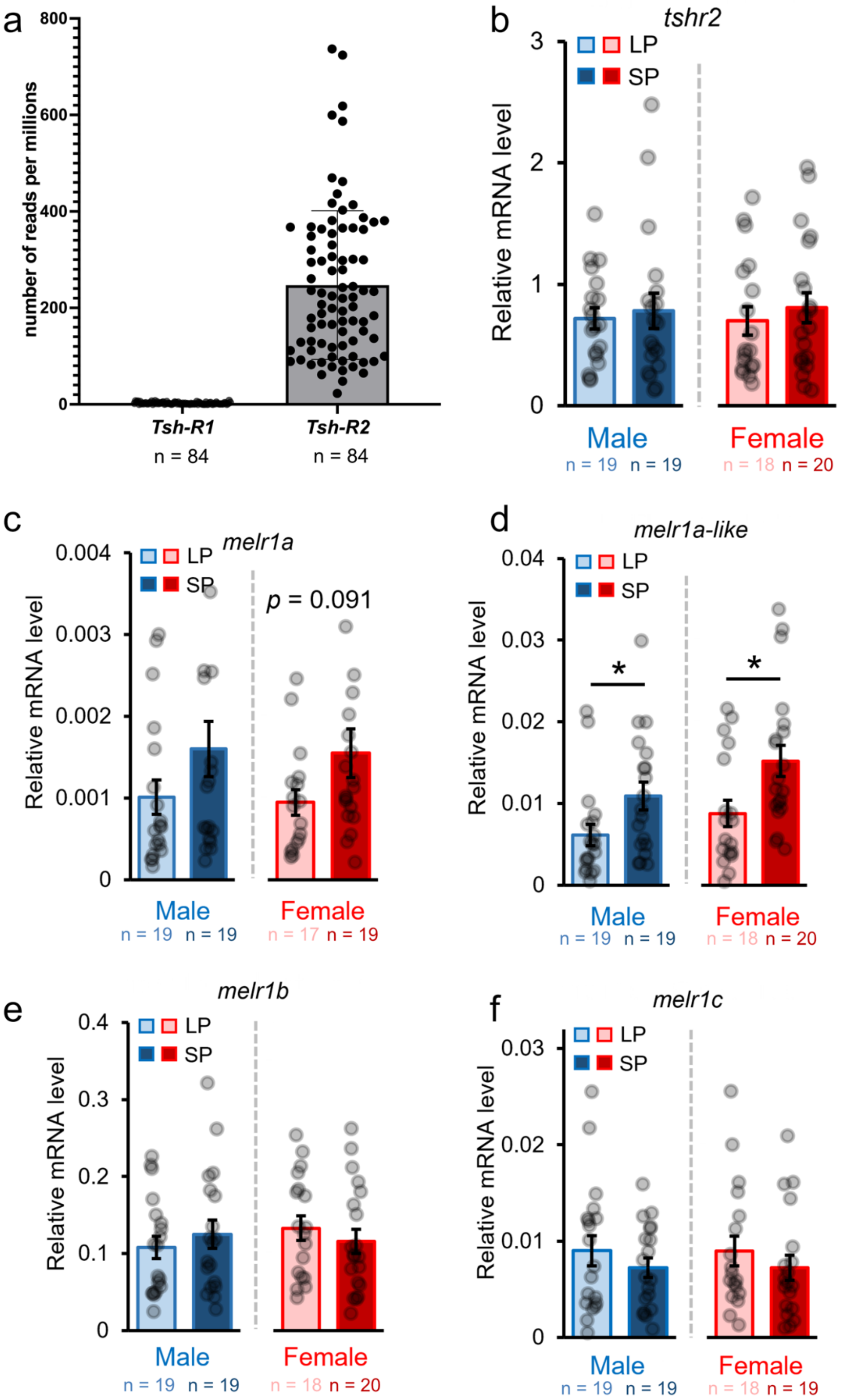

Supplementary Figure 5. One of the two identified Tsh receptors (tshr2) is expressed in the medaka pituitary with photoperiod-independent expression. (a) Number of reads per million of two Tsh receptors (arbitrarily named *tshr1* and *tshr2)* in the medaka pituitary from RNA transcriptomic data from both sexes

combined. The graph represents mean ± SEM while the jittered dots represent each individual. (b) The mRNA levels of *tshr2* in the male and female medaka pituitary in SP and LP condition. (c-d) The relative mRNA levels four melatonin receptor paralogs in the medaka pituitary, *melr1a* (a), *melr1a-like* (b), *melr1b* (c), and *melr1c* (d) from SP and LP condition. The statistical analyses were performed using two-sample independent t-test or Mann Whitney U test, in which the graph represents mean ± SEM while jitter dots represent each individual (* < 0.05). Individual numbers (n) are annotated throughout the figure.

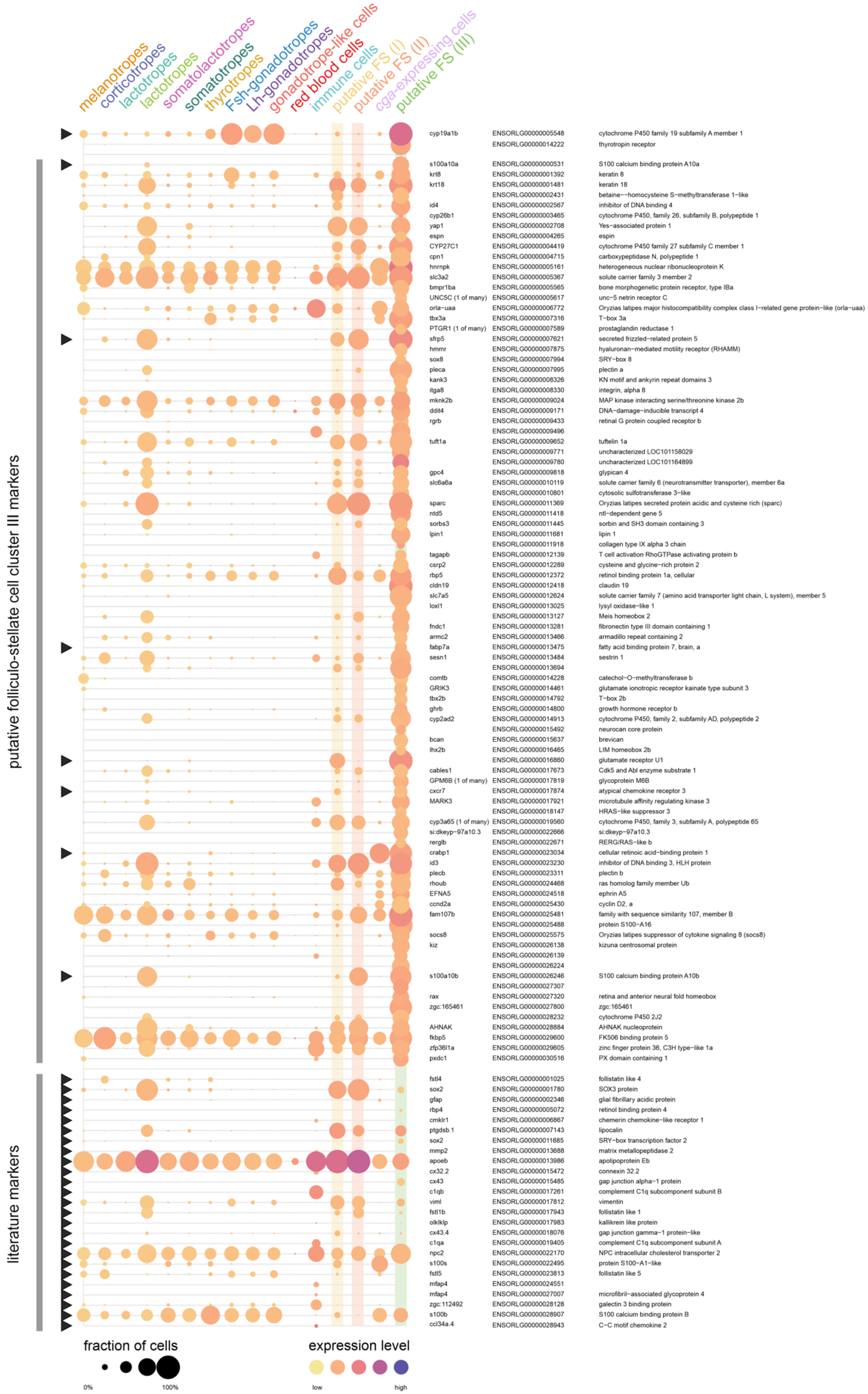

melanotropes
corticotropes
lactotropes
lactotropes
somatolactotropes
somatotropes
thyrotropes
Fsh-gonadotropes
Lh-gonadotropes
gonadotrope-like cells
red blood cells
immune cells
putative FS (I)
putative FS (II)
cga-expressing cells
putative FS (III)
putative folliculo-stellate cell cluster III markers
literature markers
cyp19a1b ENSORLG00000005548 cytochrome P450 family 19 subfamily A member 1
ENSORLG00000014222 thyrotropin receptor
s100a10a ENSORLG00000000531 S100 calcium binding protein A10a
krt8 ENSORLG00000001392 keratin 8
krt18 ENSORLG00000001481 keratin 18
ENSORLG00000002431 betaine--homocysteine S-methyltransferase 1-like
id4 ENSORLG00000002567 inhibitor of DNA binding 4
cyp26b1 ENSORLG00000003465 cytochrome P450, family 26, subfamily B, polypeptide 1
yap1 ENSORLG00000002708 Yes-associated protein 1
espn ENSORLG00000004265 espin
CYP27C1 ENSORLG00000004419 cytochrome P450 family 27 subfamily C member 1
cpn1 ENSORLG00000004715 carboxypeptidase N, polypeptide 1
hnrnpk ENSORLG00000005161 heterogeneous nuclear ribonucleoprotein K
slc3a2 ENSORLG00000005367 solute carrier family 3 member 2
bmpr1ba ENSORLG00000005565 bone morphogenetic protein receptor, type IBa
UNC5C (1 of many) ENSORLG00000005617 unc-5 netrin receptor C
orla-uaa ENSORLG00000006772 Oryzias latipes major histocompatibility complex class I-related gene protein-like (orla-uaa)
tbx3a ENSORLG00000007316 T-box 3a
PTGR1 (1 of many) ENSORLG00000007589 prostaglandin reductase 1
sfrp5 ENSORLG00000007621 secreted frizzled-related protein 5
hmmr ENSORLG00000007875 hyaluronan-mediated motility receptor (RHAMM)
sox8 ENSORLG00000007994 SRY-box 8
pleca ENSORLG00000007995 plectin a
kank3 ENSORLG00000008326 KN motif and ankyrin repeat domains 3
itga8 ENSORLG00000008330 integrin, alpha 8
mknk2b ENSORLG00000009024 MAP kinase interacting serine/threonine kinase 2b
ddit4 ENSORLG00000009171 DNA-damage-inducible transcript 4
rgrb ENSORLG00000009433 retinal G protein coupled receptor b
ENSORLG00000009496
tuft1a ENSORLG00000009652 tuftelin 1a
ENSORLG00000009771 uncharacterized LOC101158029
ENSORLG00000009780 uncharacterized LOC101164899
gpc4 ENSORLG00000009818 glypican 4
slc6a6a ENSORLG00000010119 solute carrier family 6 (neurotransmitter transporter), member 6a
ENSORLG00000010801 cytosolic sulfotransferase 3-like
sparc ENSORLG00000011369 Oryzias latipes secreted protein acidic and cysteine rich (sparc)
ntd5 ENSORLG00000011418 ntl-dependent gene 5
sorbs3 ENSORLG00000011445 sorbin and SH3 domain containing 3
lpin1 ENSORLG00000011681 lipin 1
ENSORLG00000011918 collagen type IX alpha 3 chain
tagapb ENSORLG00000012139 T cell activation RhoGTPase activating protein b
csrp2 ENSORLG00000012289 cysteine and glycine-rich protein 2
rbp5 ENSORLG00000012372 retinol binding protein 1a, cellular
cldn19 ENSORLG00000012418 claudin 19
slc7a5 ENSORLG00000012624 solute carrier family 7 (amino acid transporter light chain, L system), member 5
loxl1 ENSORLG00000013025 lysyl oxidase-like 1
ENSORLG00000013127 Meis homeobox 2
fndc1 ENSORLG00000013281 fibronectin type III domain containing 1
armc2 ENSORLG00000013466 armadillo repeat containing 2
fabp7a ENSORLG00000013475 fatty acid binding protein 7, brain, a
sesn1 ENSORLG00000013484 sestrin 1
ENSORLG00000013694
comtb ENSORLG00000014228 catechol-O-methyltransferase b
GRIK3 ENSORLG00000014461 glutamate ionotropic receptor kainate type subunit 3
tbx2b ENSORLG00000014792 T-box 2b
ghrb ENSORLG00000014800 growth hormone receptor b
cyp2ad2 ENSORLG00000014913 cytochrome P450, family 2, subfamily AD, polypeptide 2
ENSORLG00000015492 neurocan core protein
bcan ENSORLG00000015637 brevican
lhx2b ENSORLG00000016465 LIM homeobox 2b
ENSORLG00000016860 glutamate receptor U1
cables1 ENSORLG00000017673 Cdk5 and Abl enzyme substrate 1
GPM6B (1 of many) ENSORLG00000017819 glycoprotein M6B
cxcr7 ENSORLG00000017874 atypical chemokine receptor 3
MARK3 ENSORLG00000017921 microtubule affinity regulating kinase 3
ENSORLG00000018147 HRAS-like suppressor 3
cyp3a65 (1 of many) ENSORLG00000019560 cytochrome P450, family 3, subfamily A, polypeptide 65
si:dkeyp-97a10.3 ENSORLG00000022666 si:dkeyp-97a10.3
rerglb ENSORLG00000022671 RERG/RAS-like b
crabp1 ENSORLG00000023034 cellular retinoic acid-binding protein 1
id3 ENSORLG00000023230 inhibitor of DNA binding 3, HLH protein
plecb ENSORLG00000023311 plectin b
rhoub ENSORLG00000024468 ras homolog family member Ub
EFNA5 ENSORLG00000024518 ephrin A5
ccnd2a ENSORLG00000025430 cyclin D2, a
fam107b ENSORLG00000025481 family with sequence similarity 107, member B
ENSORLG00000025488 protein S100-A16
socs8 ENSORLG00000025575 Oryzias latipes suppressor of cytokine signaling 8 (socs8)
kiz ENSORLG00000026138 kizuna centrosomal protein
ENSORLG00000026139
ENSORLG00000026224
s100a10b ENSORLG00000026246 S100 calcium binding protein A10b
ENSORLG00000027307
rax ENSORLG00000027320 retina and anterior neural fold homeobox
zgc:165461 ENSORLG00000027800 zgc:165461
ENSORLG00000028232 cytochrome P450 2J2
AHNAK ENSORLG00000028884 AHNAK nucleoprotein
fkbp5 ENSORLG00000029600 FK506 binding protein 5
zfp36l1a ENSORLG00000029605 zinc finger protein 36, C3H type-like 1a
pxdc1 ENSORLG00000030516 PX domain containing 1
fstl4 ENSORLG00000001025 follistatin like 4
sox2 ENSORLG00000001780 SOX3 protein
gfap ENSORLG00000002346 glial fibrillary acidic protein
rbp4 ENSORLG00000005072 retinol binding protein 4
cmklr1 ENSORLG00000006867 chemerin chemokine-like receptor 1
ptgdsb.1 ENSORLG00000007143 lipocalin
sox2 ENSORLG00000011685 SRY-box transcription factor 2
mmp2 ENSORLG00000013688 matrix metallopeptidase 2
apoeb ENSORLG00000013986 apolipoprotein Eb
cx32.2 ENSORLG00000015472 connexin 32.2
cx43 ENSORLG00000015485 gap junction alpha-1 protein
c1qb ENSORLG00000017261 complement C1q subcomponent subunit B
viml ENSORLG00000017812 vimentin
fstl1b ENSORLG00000017943 follistatin like 1
olklklp ENSORLG00000017983 kallikrein like protein
cx43.4 ENSORLG00000018076 gap junction gamma-1 protein-like
c1qa ENSORLG00000019405 complement C1q subcomponent subunit A
npc2 ENSORLG00000022170 NPC intracellular cholesterol transporter 2
s100s ENSORLG00000022495 protein S100-A1-like
fstl5 ENSORLG00000023813 follistatin like 5
mfap4 ENSORLG00000024551
mfap4 ENSORLG00000027007 microfibril-associated glycoprotein 4
zgc:112492 ENSORLG00000028128 galectin 3 binding protein
s100b ENSORLG00000028907 S100 calcium binding protein B
ccl34a.4 ENSORLG00000028943 C-C motif chemokine 2
fraction of cells
0%
100%
expression level
low
high

Supplementary Figure 6. Genes specifically expressed in putative folliculo-stellate cell cluster III. Expression levels are indicated by colour, the percentage of number of cells in a cluster expressing a gene by circle size. At the top, the expression patterns of *tshr2* and *cyp19a1b* summarize Figure 5 b-c. Below that, an additionall 88 genes are specifically expressed in the same cells. This set includes several previously found marker genes of either folliculostellate cells* or pituicytes# (black triangles). At the bottom, homologues of other marker genes do not provide a clear identification of folliculo-stellate cells in the medaka pituitary.

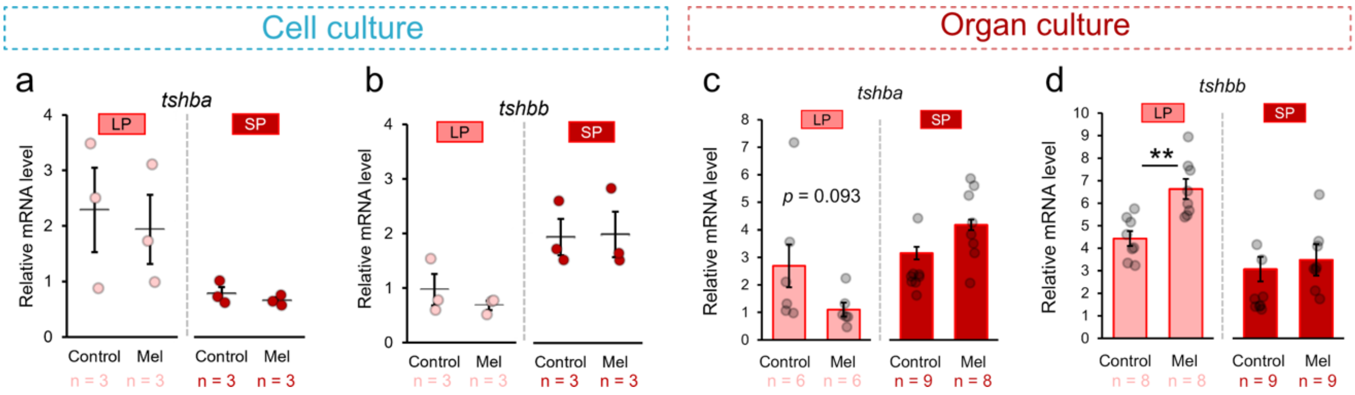

Supplementary Figure 7. Melatonin indirectly regulates Tsh cell activity. (a-b) Effect of 10 µM melatonin on *tshba* and *tshbb* levels in dispersed cell cultures of medaka pituitaries from LP and SP condition (n = 3 cultures, each n represents 4 pooled pituitaries). (c-d) Effect of 10 µM melatonin on *tshba* and *tshbb* levels in medaka pituitary organ cultures from LP and SP condition (n = 6-10). The statistical analyses were performed using two-sample independent t-test or Mann Whitney U test, in which the graphs are represented as mean ± SEM while the jittered dots represent each individual (* < 0.05; ** < 0.01). Individual numbers (n) are annotated throughout the figure.

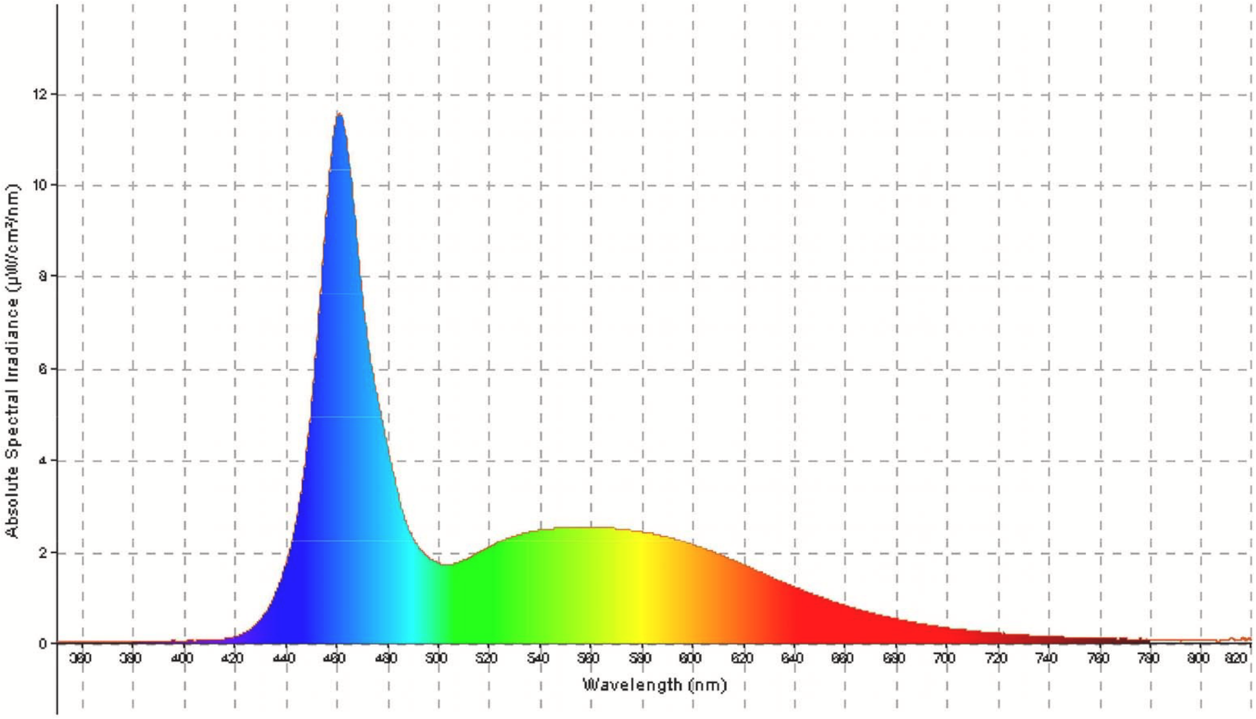

Supplementary Figure 8. Color spectrum of the light used in our study.
