# Supplementary material for: Day length regulates gonadotrope proliferation and reproduction via an intra-pituitary pathway in the model vertebrate Oryzias latipes
Source: Commun Biol. 2024 Mar 30;7:388. doi: 10.1038/s42003-024-06059-y (PMC10980775; doi:10.1038/s42003-024-06059-y)
Supplement: Supplementary file 3 — Description of Additional Supplementary Files [file 42003_2024_6059_MOESM3_ESM.pdf]

Description of Additional Supplementary Files

File name: Supplementary Data 1

Description: Contains all the numerical source data for the graphs.
