# Supplementary material for: Day length regulates gonadotrope proliferation and reproduction via an intra-pituitary pathway in the model vertebrate Oryzias latipes
Source: Commun Biol. 2024 Mar 30;7:388. doi: 10.1038/s42003-024-06059-y (PMC10980775; doi:10.1038/s42003-024-06059-y)
Supplement: Supplementary file 5 — Reporting Summary [file 42003_2024_6059_MOESM5_ESM.pdf]

Corresponding author(s): Romain Fontaine

Last updated by author(s): 2024.02.05

Reporting Summary

Nature Portfolio wishes to improve the reproducibility of the work that we publish. This form provides structure for consistency and transparency in reporting. For further information on Nature Portfolio policies, see our Editorial Policies and the Editorial Policy Checklist.

Statistics

For all statistical analyses, confirm that the following items are present in the figure legend, table legend, main text, or Methods section.

n/a	Confirmed	
☐	☒	The exact sample size (*n*) for each experimental group/condition, given as a discrete number and unit of measurement
☐	☒	A statement on whether measurements were taken from distinct samples or whether the same sample was measured repeatedly
☐	☒	The statistical test(s) used AND whether they are one- or two-sided<br>*Only common tests should be described solely by name; describe more complex techniques in the Methods section.*
☒	☐	A description of all covariates tested
☐	☒	A description of any assumptions or corrections, such as tests of normality and adjustment for multiple comparisons
☐	☒	A full description of the statistical parameters including central tendency (e.g. means) or other basic estimates (e.g. regression coefficient) AND variation (e.g. standard deviation) or associated estimates of uncertainty (e.g. confidence intervals)
☐	☒	For null hypothesis testing, the test statistic (e.g. *F*, *t*, *r*) with confidence intervals, effect sizes, degrees of freedom and *P* value noted<br>*Give P values as exact values whenever suitable.*
☒	☐	For Bayesian analysis, information on the choice of priors and Markov chain Monte Carlo settings
☒	☐	For hierarchical and complex designs, identification of the appropriate level for tests and full reporting of outcomes
☒	☐	Estimates of effect sizes (e.g. Cohen's *d*, Pearson's *r*), indicating how they were calculated

Our web collection on statistics for biologists contains articles on many of the points above.

Software and code

Policy information about availability of computer code

Data collection	Processed single-cell and regular RNA-seq data were retrieved from https://identifiers.org/geo:GSE162787 and https://doi.org/10.18710/HTCXRN , respectively.
Data analysis	Data were processed in R (version 4.0.3) using the code available at https://github.com/christiaanhenkel/medakapituitary

For manuscripts utilizing custom algorithms or software that are central to the research but not yet described in published literature, software must be made available to editors and reviewers. We strongly encourage code deposition in a community repository (e.g. GitHub). See the Nature Portfolio guidelines for submitting code & software for further information.

Data

Policy information about availability of data

All manuscripts must include a data availability statement. This statement should provide the following information, where applicable:

- Accession codes, unique identifiers, or web links for publicly available datasets
- A description of any restrictions on data availability
- For clinical datasets or third party data, please ensure that the statement adheres to our policy

All datasets generated during and/or analyzed during the current study are present in this manuscript or have been previously published.

Research involving human participants, their data, or biological material

Policy information about studies with human participants or human data. See also policy information about sex, gender (identity/presentation), and sexual orientation and race, ethnicity and racism.

Reporting on sex and gender	N/A
Reporting on race, ethnicity, or other socially relevant groupings	N/A
Population characteristics	N/A
Recruitment	N/A
Ethics oversight	N/A

Note that full information on the approval of the study protocol must also be provided in the manuscript.

Field-specific reporting

Please select the one below that is the best fit for your research. If you are not sure, read the appropriate sections before making your selection.

☒ Life sciences ☐ Behavioural & social sciences ☐ Ecological, evolutionary & environmental sciences

For a reference copy of the document with all sections, see nature.com/documents/nr-reporting-summary-flat.pdf

Life sciences study design

All studies must disclose on these points even when the disclosure is negative.

Sample size	The sample size (3 at minimum) was based on at least the required minimum sample size for statistical analysis.
Data exclusions	Not applicable
Replication	All data generated were replicated at least once and were reproducible, except for the qPCR for gnrh1 and kiss1 (Figure 1F-G) were not replicated because the experimenter has changed affiliation to an institution in another country, making it impossible to replicate the experiment.
Randomization	All the experimental animals that were in holding tanks were distributed randomly into specified groups.
Blinding	Sample collection was performed using systematic randomization.

Reporting for specific materials, systems and methods

We require information from authors about some types of materials, experimental systems and methods used in many studies. Here, indicate whether each material, system or method listed is relevant to your study. If you are not sure if a list item applies to your research, read the appropriate section before selecting a response.

Materials & experimental systems

n/a	Involved in the study	
☐	☒	Antibodies
☒	☐	Eukaryotic cell lines
☒	☐	Palaeontology and archaeology
☐	☒	Animals and other organisms
☒	☐	Clinical data
☒	☐	Dual use research of concern
☒	☐	Plants

Methods

n/a	Involved in the study	
☒	☐	ChIP-seq
☒	☐	Flow cytometry
☒	☐	MRI-based neuroimaging

Antibodies

Antibodies used	Antobodies used are described in supplemental table 2:<br>Rat anti-BrdU(abcam ab6326)

	Goat anti-rabbit Alexa-555(Invitrogen A21429)<br>Donkey anti-rat Alexa-647 (Invitrogen (A78947)<br>Custom made Rabbit anti-medakaFshβ (previously published)
Validation	These antibodies have been previously used and validated:<br>R. Fontaine, E. Ager-Wick, K. Hodne, F.-A. Weltzien, Plasticity of Lh cells caused by cell proliferation and recruitment of existing cells. Journal of Endocrinology 240, 361 (2019).<br>S. Burow et al., Medaka follicle-stimulating hormone (Fsh) and luteinizing hormone (Lh): Developmental profiles of pituitary protein and gene expression levels. General and Comparative Endocrinology 272, 93-108 (2019).<br>K. Hodne, R. Fontaine, E. Ager-Wick, F.-A. Weltzien, Gnrh1-Induced Responses Are Indirect in Female Medaka Fsh Cells, Generated Through Cellular Networks. Endocrinology 160, 3018-3032 (2019).

Animals and other research organisms

Policy information about studies involving animals; ARRIVE guidelines recommended for reporting animal research, and Sex and Gender in Research

Laboratory animals	Japanese medaka (Oryzias latipes). Adult fish (>3 months old fish) were used
Wild animals	N/A
Reporting on sex	Both sexes were used
Field-collected samples	N/A
Ethics oversight	BrDU experiments were approved by Mattilsynet (FOTS#24305)

Note that full information on the approval of the study protocol must also be provided in the manuscript.
